# The Prevalence of Metabolic Syndrome and Factors Associated with Quality of Dialysis among Hemodialysis Patients in Southern Taiwan

**DOI:** 10.5539/gjhs.v4n5p53

**Published:** 2012-07-18

**Authors:** Shu-Fen Tu, Yu-Ching Chou, Chien-An Sun, Shu-Chun Hsueh, Tsan Yang

**Affiliations:** 1 Chen Xiangguo joint clinics, Tainan, Taiwan; 2 School of Public Health, National Defense Medical Center, Taipei City, Taiwan; 3 Department of Public Health, College of Medicine, Fu Jen Catholic University, New Taipei City, Taiwan; 4 Department of Health Business Administration, Meiho University, Pingtung, Taiwan

**Keywords:** hemodialysis, metabolic syndrome, quality of dialysis

## Abstract

**Objectives:**

The purpose of this study was to evaluate the prevalence of metabolic syndrome (MetS) among hemodialysis patients and factors associated with quality of dialysis.

**Methods:**

Data were collected from 377 long-term hemodialysis patients who received hemodialysis treatment from clinics in Tainan and Kaohsiung between November 2009 and February 2010. MetS was defined using criteria set by the adult treatment panel III (ATP-III). But, the cutpoint of waist circumference has been modified to adjust for Asian populations.

The measurement of Kt/V was used as an indicator of the quality of dialysis. A below 1.4 Kt/V was considered poor dialysis quality.

**Results:**

Results showed that the prevalence of MetS among the chronic hemodialysis patients in this sample was 61.0%. Logistic regression results identified that the quality of dialysis in females was better than that in males (odds ratio (OR)=7.98, 95% confidence interval (CI): 2.52-25.31). Better quality dialysis was associated with older age, longer treatment time, and increased blood flow rate (OR=1.49, 13.63, and 1.35, respectively). However, for every one kilogram increase in weight, the quality of dialysis decreased by 13 percents (OR=0.87, 95% CI: 0.83-0.92).

**Conclusions:**

MetS is common among hemodialysis patients. The prevalence of hypertension, hyperlipidemia, and hyperglycaemia were significantly higher among hemodialysis patients. Quality of dialysis related to gender, age, weight, and the dialysis prescription (treatment time and blood flow rate).

## 1. Introduction

Cardiovascular diseases (CVDs) are common among chronic hemodialysis patients, representing the major cause of death: 44% of the total mortality in dialysis patients (Herzog, Ma, & Collins, 1998). In a study examining 627,983 long-term dialysis patients from The United States Renal Data System, of the 34,189 hospitalizations because of myocardial infarction between 1977 and 1995, 73% of the patients died following myocardial infarction, and the mortality rate in five years was nearly 90% (Herzog et al., 1998). Prevention of CVD among chronic kidney patients is, therefore, crucial. This statistic does not include end-stage renal disease (ESRD) because 30% of chronic kidney patients have already shown signs of ischaemic heart disease or heart failure before reaching this stage. Therefore, prevention and treatment of CVD are equally important in early chronic kidney disease (CKD) and ESRD (Sarnak, 2003).

Metabolic syndrome (MetS) is an independent predictor of coronary artery diseases, thrombus, and cerebral vascular disease (Najjar, El Gamal, Halabi, & Leyenson, 2005), and recognized as the major indicator of CVD occurrence and its mortality rate (Trevisan, Liu, Bahsas, & Menotti, 1998; Isomaa et al., 2001). Epidemiological studies have suggested that the risk of cardiovascular and cerebrovascular disease in patients with MetS was 38% higher than in those without MetS. CVD patients with MetS were also 1.78 times more likely to die than those without MetS (Scuteri, Najjar, Morrell, & Lakatta, 2005; Gami et al., 2007).

Although the main cause of death in CKD patients is CVD, complications of MetS increase the risk of mortality. In previous studies, the prevalence of MetS was 41.7% among CKD patients (Lea et al., 2008) and 37.7 to 69.3% among hemodialysis patients (Young, Lund, Haynatzki, & Dunlay, 2007; Lucove, Vupputuri, Heiss, North, & Russell, 2008). In one study in the United States, the prevalence of MetS in uremic patients was 69.3% (Young et al., 2007). In Taiwan, little is known regarding the prevalence of MetS in hemodialysis patients due to lack of research data. A study conducted in Eastern Taiwan identified that 49.4% of the sampled dialysis patients also had MetS (Wang, Fang, Shu, Lin, & Shen, 2009). Existing data from the United States and Eastern Taiwan, therefore, show a high positive correlation between CKD and MetS. The results from a study by Wolfe et al. indicated that the quality of hemodialysis treatment (Kt/V) is an independent risk factor relating to mortality among hemodialysis patients, and that there was a negative correlation between quality of dialysis and mortality rate (Wolfe, Ashby, Daugirdas, Agodoa, Jones, & Port, 2000). The factors affecting quality of dialysis include gender, body size [such as weight, body mass index (BMI), and body surface area] and dialysis dose (blood flow rate, treatment time, and the membrane area of the artificial kidney) (Molina et al., 2010). Kt/V closely relates to the urea reduction ratio. A higher Kt/V value indicates greater hemodialysis efficiency or greater excretion of wastes in blood. The Kidney Disease Outcomes Quality Initiative (K/DOQI) guidelines of the United States recommended a Kt/V of 1.2 for patients with three treatments per week. The NKF-DOQI clinical practice guidelines for hemodialysis adequacy, however, recommended a Kt/V of 1.3 (NKF-DOQI, 1997). The study of Held et al. indicated that every 0.1 increase in Kt/V can reduce the death risk by 7%, and every 5% increase in urea reduction ratio (URR) can reduce the death risk by 11% (Held et al., 1996). Few studies in Taiwan have examined the factors affecting the quality of dialysis. For these reasons, the present study was aiming at evaluating the prevalence of MetS in hemodialysis patients in Southern Taiwan, and also analyzing the factors affecting the quality of dialysis.

## 2.Methods

This was a cross-sectional study, following a research protocol approved by the Institutional Review Board at Meiho University, Pingtung, Taiwan. Participants were long-term hemodialysis patients recruited from the hemodialysis clinics in Tainan and Kaohsiung. These subjects were 18 years old and over, literate, and fluent in Mandarin or Taiwanese. All subjects were diagnosed with stage 5 CKD with over three months of hemodialysis treatment in dialysis clinics. Their costs of dialysis were paid for by the National Health Insurance (NHI), without copayments. Subjects were excluded if they were unconscious, or had mental disorders or metastatic cancer. This study was conducted from November 2009 to February 2010, and 382 hemodialysis patients participated in the survey. Five individuals were excluded because of incomplete information on physical and biochemical blood tests, leaving 377 patients in the current analysis.

In the present study, long-term hemodialysis patients were indicated as patients with chronic renal failure who obtained NHI copayment-waived certification of serious diseases received hemodialysis treatment in hemodialysis clinics for more than three months with a regular three sessions per month, each session lasting for two to five hours. In addition, Kt/V is by far the most commonly used marker for dialysis quality, where “K” represents the dialyzer clearance rate for urea and body water removal (ml/min or L/h), “t” represents the treatment time (min or h), and “V” refers to the urea distribution volume (ml or L) (Gotch, 2000; Kooman, van der Sande, & Leunissen, 2001). The Taiwan Society of Nephrology applies a dialysis quality of 1.3 as the cut-off point during dialysis assessment. Because of the limited number of participants with a dialysis quality of less than 1.3, the present study adopted a more rigorous dialysis quality standard of 1.4. A Kt/V of less than 1.4 indicated poorer dialysis quality. Furthermore, the definition of Mets was based on the the criteria set in the Third Report of the National Cholesterol Education Program (NCEP) Expert Panel on Detection, Evaluation, and Treatment of High Blood Cholesterol in Adults (Adult Treatment Panel III, ATP-III) (“Executive Summary of The Third Report of The National Cholesterol Education Program (NCEP) Expert Panel on Detection, Evaluation, And Treatment of High Blood Cholesterol In Adults (Adult Treatment Panel III),” 2001). We utilized previously established modifications for Asian populations, using waist circumference cut-points (Tan, Ma, Wai, Chew, & Tai, 2004). Any three of the following five criteria were grounds for identifying metabolic syndrome: (1) abdominal obesity: waist circumference (WC) ≥90 cm in men and ≥80 cm in women; (2) raised triglyceride (TG): ≥150 mg/dL; (3) reduced high-density lipoprotein cholesterol (HDL-C): HDL-C <40 mg/dL in men and <50 mg/dL in women; (4) hypertension: blood pressure of at least 130/85 mm Hg or taking antihypertensive medication; and (5) raised fasting plasma glucose (FPG) ≥110 mg/dL and/or taking anti-glycemic medication.

## 3. Data Collection

A self-administered questionnaire was used to collect information on participants’ sociodemographic characteristics, medical history, and hemodialysis prescription including dialysis class, dialysis time, flow rate, and membrane area of the artificial kidney. Blood pressure and anthropometric measurements, including height, weight, and WC, were also taken. Height was measured to the nearest 0.1 cm, without shoes, using a stadiometer (Model No.: Hold Cheer BW-120). Weight was measured in light clothing, without shoes, using a beam balance scale, and was recorded to the nearest 0.1 kg. WC was measured to the nearest 0.5 cm above the iliac crests and below the lowest rib margin at minimal respiration in a standing position. Each participant’s blood pressure measurements were obtained using a standardized protocol. Blood pressure was measured by nurses trained to treat hemodialysis patients within 5 to 10 minutes of the start of dialysis treatment. The participant placed the wrist on the bed, palm facing upward, maintaining the arm and heart at the same height. Blood pressure measurements were taken twice using an electronic automatic blood pressure device (model 3M Littmann Classic 2 S. Stethoscope). If the readings varied by more than 10 mmHg, an additional measurement was taken. The average of the two closest readings of blood pressures was used in the analysis. Fasting venous blood samples were collected from each subject for a battery of biochemistry analyses. The study subjects had fasted for 12 hours prior to blood collection. Each participant had 12 ml of blood drawn, including one 5-ml sample for biochemical examination, one 3-ml sample for routine blood analysis, and one 2-ml sample for glucose analysis. These blood specimens were retained prior to dialysis treatment, and one further 2-ml of blood was collected for biochemical examination before the end of dialysis treatment. This sample was used to examine the urea nitrogen after dialysis treatment. After entering the examination data into a computer, the Kt/V could be calculated to evaluate the quality of dialysis.

## 4. Statistical Analysis

Mean± standard deviation (SD), frequency, and percentages were used to describe the characteristics of the study subjects. Independent *t*-test was performed to compare the mean differences in continuous variables between quality of dialysis (good and poor group). Multiple logistic regression was used to estimate the odds ratio for predicted factors affecting dialysis quality. All statistical tests were two-tailed, and values of p<0.05 were considered statistically significant. Data were analyzed using SPSS for Windows, version 17.0 (SPSS Inc., Chicago, IL, USA).

## 5.Results

As shown in Table 1, study subjects had an average age of 64.13±12.24 years. Females comprised 203 (53.8%) participants. Results from anthropometric examinations identified that the average BMI was 22.75 ± 3.96 kg/m^2^, and BMI did not exceed 27 kg/m^2^ in most of the study subjects. The average WC was 90.78 ± 10.19 cm. A total of 272 (72.1%) subjects had abdominal obesity, and 84.4% of the participants suffered from hypertension. The prevalence rates for hyperglycemia, reduced HDL-C, and hypertriglyceridemia were 53.1%, 53.8%, and 36.1%, respectively. The prevalence of MetS was 61.0%. One hundred and seventy-seven (46.9%) participants received dialysis because of chronic glomerulonephritis, 55 (14.6%) participants because of chronic interstitial nephritis or hypertensive nephropathy, and 117 (31.0%) participants because of nephropatia diabetes.

**Table 1:** Sociodemographic and clinical characteristics of the study participants

Variables	Number (%)	Mean±SD
**Characteristic**		
**Age**		64.13±12.24
65yr	191(50.7%)	
≥65yr	186(49.3%)	
**Sex**		
Male	174(46.2%)	
Female	203(53.8%)	
**Education level**		
No	77(20.4%)	
Primary school	139(36.9%)	
Junior high school	70(18.6%)	
High school	71(18.8%)	
Academy	11(2.9%)	
College	9(2.4%)	
**Job**		
Yes	62(16.4%)	
No	315(83.6%)	
**Metablic syndrome components**		
Height(cm)		159.12±9.24
Weight(kg)		57.66±11.20
**Body mass index(kg/m^^2^^)**		22.75±3.96
BMI <27	335(88.9%)	
BMI ≥27	42(11.1%)	
**Waist circumference(cm)**		90.78±10.19
Normal	105(27.9%)	
Abnormal^a^	272(72.1%)	
Hypertension(mmHg)^b^	318(84.4%)	
**Hyperglycemia(mg/dL)^c^**	200(53.1%)	
Low HDL-C(mg/dL)^d^	203(53.8%)	
Hypertriglyceridemia^e^	136(36.1%)	

**Number of metabolic syndrome indicators**		
0	2(0.5%)	
1	46(16.2%)	
2	99(26.3%)	
3	91(24.1%)	
4	83(22.0%)	
5	56(14.9%)	
**Metabolic syndrome**		
Not present	147(39.0%)	
Present^f^	230(61.0%)	
**Reason of dialysis**		
Cronic glomerulonephritis	177(46.9%)	
Cronic interstitial nephritis or hypertensive nephropathy	55(14.6%)	
Nphropatia diabetic	117(31.0%)	
Other	28(7.4%)	
**Dalysis treatment**		
Dialysis duration(yrs)		5.71±3.99
**Fstula type of hemodialysis**		
Atologous	313(83.0%)	
Atificial	64(17.0%)	
Dialysis treatment time(h)		3.99±0.21
Area of artificial kidney(m^2^)		1.83±0.24
Blood flow rate(cc/min)		284.06±39.85

a Male: ≥90 cm; female: ≥80 cm.

b ≥130/85 mmHg or taking antihypertensive medication.

c ≥110 mg/dL or taking medication for lowering blood glucose.

d HDL-C: high-density lipoprotein cholesterol: Male:<40 mg/dL; female<50 mg/dL.

e ≥150 mg/dL.

f Metabolic syndrome definition of NCEP ATP III with modification for Asian populations

The average duration of dialysis in the study subjects was 5.71 ± 3.99 years (range: less than 1 to 22 years). 313 (83.0%) subjects received autologous fistula for hemodialysis, and 64 (17.0%) subjects received artificial fistula. Each dialysis treatment session lasted an average of 3.99 ± 0.21 hours; more than four hours in 354 (93.9%) subjects and less than four hours in 23 (6.1%) subjects. The smallest membrane area of the artificial kidney was 1.3 m^2^, the largest area was 2.5 m^2^, and the average was 1.83 ± 0.24 m^2^. The lowest flow rate of each dialysis was 170 mL/min, the highest was 350 mL/min, and the average was 284.1 ± 39.9 mL/min.

Table 2 displays the laboratory analyses data of hemodialysis patients. FPG, HDL-C, and uric acid levels had the highest percentages of abnormal values; 48.3%, 53.8%, and 58.1%, respectively.

**Table 2:** Laboratory analyses data of hemodialysis patients

Variables	Mean	SD	Normal n (%)	Abnormal n (%)	Definition of abnormal
Pre-dialysis BUN(mg/dL)	65.37	15.46			
post-dialysis BUN(mg/dL)	17.39	11.82			
Ca(mg/dL)	9.73	0.76	279(74.0%)	98(26.0%)	>10.1
P(mg/dL)	5.04	1.32	255(67.6%)	122(32.4%)	>5.5
K(mmol/L)	4.85	0.68	246(65.3%)	131(34.7%)	>5
Hemoglobin(g/dL)	10.90	1.17			
Hemotocrit(%)	33.25	3.78	315(83.6%)	62(16.4%)	<30
Fasting plasma glucose (mg/dL)	126.03	57.69	195(51.7%)	182(48.3%)	≥110
HDL-C(mg/dL)	46.07	13.58	174(46.2%)	203(53.8%)	male<40 or female<50
Triglyceride(mg/dL)	143.67	82.72	241(63.9%)	136(36.1%)	≥150
Total cholesterol(mg/dL)	177.96	36.24	277(73.5%)	100(26.5%)	>200
Albumin(g/dL)	3.93	0.27	249(66.0%)	128(34.0%)	≤3.8
Alk-P(U/L)	88.94	51.42	330(87.5%)	47(12.5%)	>140
Uric acid(mg/dL)	7.45	1.38	158(41.9%)	219(58.1%)	>7
GOT(U/L)	24.92	29.57	343(91.0%)	34(9.0%)	>38
GPT(U/L)	23.92	37.72	339(89.9%)	38(10.1%)	>40

The present study used a dialysis quality (Kt/V) of 1.4 as the cut-off point to divide the study subjects into two groups. Non-significant associations were found between metabolic syndrome-related indicators and hemodialysis quality (see table 3). Table 4 shows the differences between the groups on characteristics and laboratory analyses data. Patients with higher dialysis quality tended to be older and had shorter duration dialysis treatment time than patients with poor dialysis quality (p<0.001 and p=0.017, respectively). Height, weight, WC, and BMI were significantly higher in the hemodialysis patients with poor dialysis quality than those with higher dialysis quality (p<0.001). The hemodialysis patients with poor dialysis quality showed significantly higher average values for membrane area of the artificial kidney, blood flow rate, and systolic and diastolic blood pressures compared to patients with higher dialysis quality (p<0.001, p=0.001, p=0.011, and p<0.001, respectively). Results from laboratory examinations indicated that the blood urea nitrogen and albumin levels after dialysis were significantly higher in hemodialysis patients with poor dialysis quality than in patients with higher dialysis quality (p<0.001 and p=0.018, respectively). HDL-C was lower in hemodialysis patients with poor dialysis quality than in patients with higher dialysis quality (p=0.004).

**Table 3:** The differences of dialysis quality in the metabolic syndrome and and its components

Variables	Quality of dialysis (Daugirdas KT/V)≤1.4. n=65, (5.3%)	Quality of dialysis (Daugirdas KT/V)> 1.4. n=312(94.7%)	P^*^ value
Metabolic syndrome			.350
Not present	22(15.0%)	125(85.0%)	
Present^a^	43(18.7%)	187(81.3%)	
Waist circumference(cm) Normal			.975
Normal	18(17.1%)	87(82.9%)	
Abdominal obesity^b^	47(17.3%)	225(82.7%)	
Triglyceride(mg/dL)			.313
Normal	38(15.8%)	203(84.2%)	
Hypertriglyceridemia^c^	27(19.9%)	109(80.1%)	
HDL-C(mg/dL)			.274
Normal	26(14.9%)	148(85.1%)	
Low HDL-C^d^	39(19.2%)	164(80.8%)	
Blood Pressure(mmHg)			.117
Normal	6(10.2%)	53(89.8%)	
Hypertension^e^	59(18.6%)	259(81.4%)	
Fasting plasma glucose (mg/dL)			.498
Normal	33(18.6%)	144(81.4%)	
Hyperglycemia^f^	32(16.0%)	168(84.0%)	

a Metabolic syndrome definition of NCEP ATP III with modification for Asian populations.

b Male: ≥90cm; female:≥80cm.

c ≥150 mg/dL.

d Male:<40 mg/dL; female<50 mg/dL.

e ≥130/85mmHg or taking antihypertensive medication.

f ≥110 mg/dL or taking medication to lower blood glucose.

* The Chi-square (two-tailed) test was used to compare the variables, with a significance level of α=.05

**Table 4:** The differences of dialysis quality in the subjects’ demographic characteristics and laboratory analyses data

Variables	Quality of dialysis (Daugirdas KT/V)≤1.4 n=65, (5.3%)	Quality of dialysis (Daugirdas KT/V)> 1.4 n=312 (94.7%)	P* value
Age(yrs)	58.31±12.57	65.34±11.83	<.001
Height(cm)	166.60± 7.35	157.76± 8.04	<.001
Weight(kg)	69.20±11.62	55.26± 9.50	<.001
WC(cm)	96.16±10.63	89.66± 9.74	<.001
BMI(kg/m^2^)	24.94± 3.90	22.29± 3.82	<.001
Dialysis duration(yrs)	4.75± 3.35	5.91± 4.10	.017
Dialysis treatment time(hr)	4.01± 0.35	3.99± 0.17	.697
Area of artificial kidney(m^2^)	2.00± 0.24	1.79± 0.23	<.001
Blood flow rate(cc/min)	297.85±30.49	282.45±35.65	.001
Systolic blood pressure(mmHg)	150.57±24.71	142.69±22.33	.011
Diastolic blood pressure(mmHg)	83.02±12.96	77.01±10.48	<.001
Ca(mg/dL)	9.60± 0.79	9.75± 0.75	.154
P(mg/dL)	5.31± 1.51	4.99± 1.28	.071
Ca x P(mg/dL)^2^	51.17±15.45	48.89±14.16	.245
K	4.78± 0.67	4.86± 0.68	.367
Pre-dialysis BUN(mg/dL)	68.73±19.92	64.67±14.30	.122
post-dialysis BUN(mg/dL)	23.93± 9.22	16.03±11.86	<.001
Fasting plasma glucose (mg/dL)	123.82±62.01	126.49±56.83	.734
Hemotocrit(%)	33.65± 4.34	33.17± 3.65	.356
HDL-C(mg/dL)	41.65±11.76	46.99±13.77	.004
Triglyceride(mg/dL)	152.09±98.49	141.91±79.11	.367
Total cholesterol(mg/dL)	173.17±32.01	178.96±37.03	.242
Uric acid(mg/dL)	7.60± 1.39	7.42± 1.38	.334
Albumin(g/dL)	4.02± 0.32	3.91± 0.25	.018
GOT(U/L)	20.11± 9.82	25.93±32.11	.149
GPT(U/L)	19.92±13.32	24.75±40.98	.349

* Independent t-test was used to compare the variables and adopted two-tailed test, the significant level α=0.05

Multiple logistic regression analysis was used to examine the predictive factors for quality of dialysis. Potential factors for the model included age, gender, height, weight, dialysis treatment time, area of artificial kidney, blood flow rate, systolic and diastolic blood pressures, uric acid, and blood urea nitrogen before dialysis (Table 5). Results showed that, for every ten years’ increase in age, there was a 1.49 times higher probability of receiving higher dialysis quality (Kt/V>1.4) than poor dialysis quality (Kt/V≤1.4) (95% CI: 1.06-2.01). The quality of dialysis was higher in females than in males (OR=7.98, 95% CI: 2.52-25.31). Each 1 kg increase in body weight, the quality of dialysis decreased by 13 percents (OR=0.87, 95% CI: 0.83-0.92).

**Table 5:** Multiple logistic regression analysis of the predictors for quality of dialysis

Variables	β	OR(95%CI)*	p-vale
Age (yrs)	0.40	1.49(1.06- 2.01)	.021
Gender^a^	2.08	7.98(2.52-25.31)	<.001
Height(cm)	-0.01	0.99(0.93- 1.06)	.853
Weight(kg)	-0.13	0.87(0.83- 0.92)	<.001
Dialysis treatment time(hr)	2.61	13.63(2.51-74.01)	.002
Area of artificial kidney(m^2^)	-1.20	0.30(0.04- 2.12)	.227
Blood flow rate(cc/min)	0.30	1.35(1.04- 1.74)	.023
Systolic blood pressure(mmHg)	0.16	1.17(0.95- 1.45)	.148
Diastolic blood pressure(mmHg)	-0.38	0.68(0.43- 1.01)	.094
Uric acid(mg/dL)^b^	0.81	2.24(0.96- 5.23)	.063
Pre-dialysis BUN(mg/dL)	-0.02	0.98(0.96- 1.01)	.292
Constant	-3.41		.624

Note: Dependent variable: quality of dialysis (Kt/V), using the ≤1.4 group as the reference group

* OR (95% CI)=odds ratio (95% confidence interval)

a Male as the reference group

b Using the uric acid≤7 as the reference group

Every one hour increase in dialysis treatment time increased the probability of receiving higher dialysis quality than poor dialysis quality by 13.63 times (95% CI: 2.51-74.01). Each 20 ml/min increase in blood flow rate contributed to a higher dialysis quality (OR=1.35, 95% CI: 1.02-1.32). The other variables did not significantly influence the quality of dialysis.

## 6.Discussion

In the general population, the prevalence of MetS is less than 30%. For instance, prior research has identified prevalence rates of 20.4% in Latin America (Escobedo et al., 2009), 26.9% in Japan (Miyatake, Kawasaki, Nishikawa, Takenami, & Numata, 2006), and 24.8% in South Korea (Kwon et al., 2005). In the event of diseases, the prevalence of MetS is considerably higher. For example, in previous studies the prevalence of mental disease with MetS was 34.2% (Kim et al., 2010), and the prevalence of heart disease with MetS was 53.2% (Yoo, Jeong, Park, Kang, & Ahn, 2009). The present study’s subjects consisted of patients undergoing long-term hemodialysis treatment in hemodialysis clinics in Southern Taiwan. A total of 377 effective samples were used, and the prevalence of MetS was 61.0%. This figure was considerably higher than the prevalence of MetS in a hemodialysis group in Eastern Taiwan (49.4%) (Wang et al., 2009). The differences may have resulted from the different criteria applied for the diagnosis of MetS; Wang et al. used the NCEP-ATP III criteria, whereas the present study applied the criteria of the NCEP-ATP III. Waist circumference was modifed for Asian populations. The differences occurred in the criteria for WC (NCEP-ATP III criteria specified a WC of ≥102 cm in males and ≥88 cm in females) and the sample size (the study by Wang et al. had 146 subjects). Wang et al. recruited subjects from a single hemodialysis center, whereas the present study recruited subjects from four different hemodialysis clinics, and therefore, is a closer representation of the actual MetS situation. These results demonstrated that the prevalence of MetS is higher in the presence of complications of other diseases than without other disorders.

In the present study, the prevalence of hypertension, abdominal obesity, hyperglycemia, reduced HDL-C, and hypertriglyceridemia was 84.4%, 72.1%, 53.1%, 58.3%, and 36.1%, respectively. Other related studies provided similar results for the prevalence of hypertension (Young et al., 2007; Wang et al., 2009). Hypertension arises because of renal function insufficiency. The renal drainage ability of the hemodialysis patient decreases, and therefore, the body cannot immediately discharge excess water. The patient requires dialysis treatment to discharge the water through a dialyzer. Current dialysis treatment takes three sessions per week, each session lasting approximately 3.5 to 5 hours. Before receiving dialysis, the excess water and stagnation of sodium increase the burden on the heart, the activity of the peripheral nervous system, and total peripheral vascular resistance, leading to symptoms of hypertension (Young et al., 2007; Wang et al., 2009).

In the present study, the prevalence of abdominal obesity was 72.1%, which was higher than the other studies conducted by Wang, Young, and their colleagues (Young et al., 2007; Wang et al., 2009). The research of Sandeep et al. (2010) identified that abdominal obesity relates to insulin resistance, cardiovascular risk factors, and MetS. A study also suggested that obesity relates to comorbidity, including MetS and CVD (Mahjoub, Gamoudi, Jamoussi, Gaigi, & Blouza-Chabchoub, 2010). Cordeiro et al. (2010) conducted a study on 173 hemodialysis patients, aged 51 to 74, identifying that abdominal obesity increased the risk of death and worsens prognoses. According to the research of Young et al. (2007), individuals with MetS first became overweight or develop obesity, and then risk factors of MetS, specifically abdominal obesity, usually follow. CKD patients should carefully monitor for adverse effects associated with excess weight and obesity because these disorders could directly cause and develop into end-stage renal diseases and sudden death because of diabetes, hypertension, and CVD (Ting, Nair, Ching, Taheri, & Dasgupta, 2009).

The present study adopted multiple logistic regression analysis to examine the factors influencing the quality of dialysis. Results showed that, when adjusting for potential confounding factors, every increase of 10 years in age resulted in a 1.49 times (95% CI: 1.06-2.01) increased probability of higher dialysis quality (Kt/V>1.4) than poor dialysis quality (Kt/V≤1.4). This indicated that older patients receive higher dialysis quality. Valdes et al. also conducted a study on hemodialysis patients, revealing that patients aged older than 65 years had higher Kt/V than patients aged 65 years old and younger (Valdés, García-Mendoza, Rebollo, T. Ortega, & F. Ortega, 2006). The dyliasis quality was 7.98 times higher in females than in males (p<0.001). This result was similar to that of a previous study conducted by Kutner et al. (2005) in which, irrespective of race, quality of dialysis was higher in females than in males. Some studies observed that increasing dialysis treatment time (Termorshuizen et al., 2003), blood flow rate (Borzou, et al., 2009), and area of the artificial kidney led to increases in Kt/V (Molina et al., 2010). In the present study, increasing dialysis treatment time and blood flow rate was also postively correlated with Kt/V. Previous studies have defined Kt/V as the dialysis dose given, where K refers to the rate of removal of body urea and water by the dialyzer (ml/min or L/h), t refers to the treatment time (min or h), and V represents the urea distribution volume (ml or L) (Mahjoub et al., 2010; Miyatake et al., 2006). The urea distribution volume (V= weight multiplied by 0.58) is positively proportional to the weight and, under constant K and t, increasing V causes a reduction in Kt/V. Therefore, after controlling for these predictive factors for quality of dialysis, increasing weight negatively correlates with Kt/V. In 1999, Beige et al. suggested that BMI negatively affects Kt/V (Beige, Sharma, Distler, Offermann, & Preuschof, 1999). Weight and BMI positively correlate, and therefore, a heavier weight is related to a greater BMI value. Hence, results from the present study indirectly demonstrated the negative correlation between weight and dialysis quality.

One limitation of this study was the purposive sampling of subjects from Tainan and Kaohsiung hemodialysis clinics; therefore, results could not completely avoid selection bias. Although inferring nationwide hemodialysis trends from the study findings is impossible, the large study population enables the interpretation of the regional quality of dialysis from the results.

In conclusion, the present study identified a prevalence of MetS of 61.0% in hemodialysis patients. The majority of hemodialysis patients have a preexisting MetS condition. The dialysis quality positively correlated with dialysis prescription (treatment time and blood flow rate), and negatively correlated with weight. Therefore, incorporating strategies for MetS prevention with dialysis prescription can reduce the incidence of hemodialysis and improve dialysis quality. Preventing MetS and its risk factors, accompanied by dialysis dose adjustment, should provide an effective means of reducing the prevalence of dialysis and improve its quality.
